# Development and Validation of a Kinetics Prediction Model for Football Cutting Using a Single Trunk-Mounted IMU

**DOI:** 10.3390/s26092741

**Published:** 2026-04-28

**Authors:** Inae Kim, Soo-ji Han, Joong Hyun Ryu, Sanghyuk Han, Jinsung Yoon, Jongchul Park

**Affiliations:** 1 Department of Physical Education, Graduate School, Pukyong National University, Busan 48513, Republic of Korea; kin2936@naver.com; 2 Industry-University Cooperation Foundation, Pukyong National University, Busan 48513, Republic of Korea; soojihan@pknu.ac.kr; 3 Performance Support & Analytics Section, Aspire Academy, Doha 22287, Qatar; 4 Rehabilitation Department, Aspetar Orthopaedic and Sports Medicine Hospital, Doha 29222, Qatar; 5 Fitogether Inc., Seoul 04378, Republic of Korea; 6 Department of Marine Sports, Pukyong National University, Busan 48513, Republic of Korea

**Keywords:** cutting, inertial measurement unit, machine learning, vertical ground reaction force, joint moment

## Abstract

This study aimed to estimate vertical ground reaction force (vGRF) and lower-limb joint moments during football cutting movements using a trunk-mounted inertial measurement unit (IMU) combined with a Random Forest model, and to validate the feasibility of this approach. IMU data collected during 45° cutting tasks were corrected using an Extended Kalman Filter (EKF). The model demonstrated good and consistent performance for vGRF (coefficient of determination, *R*^2^ = 0.766; correlation coefficient, *r* = 0.796) and sagittal plane moments of the ankle and knee (*R*^2^ = 0.661–0.689, *r* = 0.807–0.842). While Bland–Altman analysis indicated low bias and generally good agreement, precision at the individual-trial level and accuracy for non-sagittal plane moments somewhat reflected the inherent within-player trial-to-trial variability in movement execution, particularly in non-sagittal loading patterns. It should be noted that performance estimates under the current trial-based validation design may differ from those obtained using a subject-independent framework such as leave-one-subject-out cross-validation. This study demonstrates that a single trunk-mounted IMU can reliably estimate key lower-limb loading patterns, providing a practical foundation for wearable-based kinetic monitoring in applied football settings.

## 1. Introduction

### 1.1. Biomechanical Loads and Injury Risks in Football Cutting Movement

Football is a sport characterized by frequent high-intensity movements such as sprinting, change-of-direction (COD), acceleration, and deceleration. Players perform more than 700 cutting maneuvers per match to evade defenders or alter attacking directions. For this reason, cutting actions are critical determinants of performance [1]. Cutting movements impose substantially greater mechanical loads on lower-limb joints. Sharper directional changes markedly increase vertical ground reaction forces (vGRF) and hip, knee, and ankle joint moments, thereby elevating injury risk [2,3]. Ankle sprains are among the most prevalent and recurrent injuries in football and frequently occur during jump landings or rapid directional changes [4]. Furthermore, cutting significantly increases knee joint loading and is a primary mechanism for anterior cruciate ligament (ACL) injuries [5]. Multiplanar loads, particularly the combined effect of knee valgus and internal rotation moments, are key biomechanical mechanisms underlying both cutting performance and ACL injury risk [3,6]. Consequently, quantifying ground reaction forces (GRF) and joint moments during cutting is essential for load management, injury prevention, and performance enhancement [7].

### 1.2. Limitations of Laboratory-Based Approaches

The gold standard for measuring GRF and lower-limb joint moments involves the use of optical motion capture systems in combination with force plates, which are typically confined to laboratory settings. While highly accurate, this approach is confined to laboratory settings, which restricts ecological validity and precludes the accurate capture of biomechanical parameters in match-like environments. Consequently, as field-based analysis remains largely restricted to kinematic variables, there is a clear need for wearable sensor-based approaches to bridge the gap between laboratory research and real-world performance [8,9].

### 1.3. Advances in Inertial Measurement Unit (IMU)-Based Prediction Models

To overcome these limitations, recent studies have increasingly combined single IMU data with machine learning techniques to estimate kinetic variables based on the mechanical relationship between body acceleration and GRF [10,11,12]. Furthermore, research is expanding from lower-limb attachments to sensor placements on the upper trunk, such as the sacrum or between the scapulae [12,13]. This contributes to increased wearer comfort and the sustainability of data collection in actual matches. For accurate estimation, it is necessary to control for IMU drift and ensure precise attitude estimation through sophisticated filtering algorithms such as the Extended Kalman Filter (EKF) [14].

While deep learning models such as Long Short-Term Memory (LSTM) networks have shown high performance in estimating kinetic variables, they necessitate large-scale datasets. In contrast, tree-based models such as Random Forest provide stable performance with relatively smaller datasets, effectively capture non-linear relationships in time-series data, and offer improved interpretability through feature importance analysis [11,15,16].

In recent years, the use of trunk-worn Electronic Performance and Tracking Systems (EPTS) has become ubiquitous for in-match data collection in football. Following the official permission for their use in competitive matches, these systems integrate Global Navigation Satellite Systems (GNSS), IMU, and heart rate (HR) sensors to monitor player activity in real time [17,18]. This suggests the possibility of implementing laboratory-grade kinetic analysis in actual match environments by utilizing the IMUs included in the EPTS currently used in football settings worldwide. However, existing studies have not simultaneously addressed high-speed cutting tasks, single-sensor practicality compatible with commercial EPTS, and stable orientation estimation under extreme dynamic conditions. The present study therefore extends this line of research by targeting all three dimensions.

### 1.4. Study Objectives

This study aimed to extend the approach of kinetic analysis to actual sports environments by developing and validating a predictive model to estimate vGRF and lower-limb joint moments during football cutting movements using a single trunk-mounted IMU. In doing so, this research extends the current literature through three primary contributions: explicitly targeting high-speed, high-risk cutting to capture injury-related multi-axis joint moments (task specificity); utilizing a single IMU at the exact location of commercial EPTS to ensure on-field applicability (practical compatibility); and applying an EKF to enhance postural estimation during extreme dynamic movements, establishing a technical foundation for future integration with GNSS tracking (technical differentiation).

## 2. Materials and Methods

### 2.1. Participants

Thirteen healthy male amateur football players from university football clubs participated in this study (age: 23.5 ± 2.3 years; height: 175.3 ± 6.7 cm; body mass: 75.2 ± 7.1 kg; playing experience: 8.2 ± 3.0 years). Participants were right-foot dominant with a minimum of five years of football experience and had no history of lower-limb injury or musculoskeletal disorders within six months prior to data collection. The study was approved by the Institutional Review Board of Pukyong National University (IRB No. 2025-05-005), and all participants provided voluntary written informed consent prior to participation.

### 2.2. Experimental Protocol

**Figure 1:**
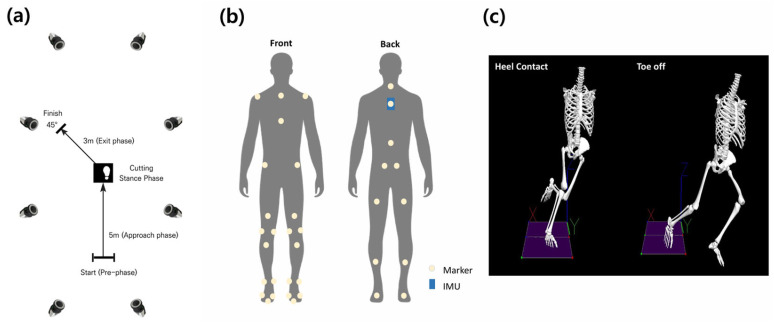
Experimental setting (**a**) Experimental setup, (**b**) Marker set, (**c**) analysis zones.

Participants completed a 5 min warm-up followed by practice trials before performing five valid 45° cutting trials. Participants approached a 5 m approach zone at 4.5 ± 0.5 m/s, planted their right foot on the force plate, and performed a left lateral cut, passing through a 3 m exit zone [16,19]. Approach speed was monitored in real time using Speed time measurement system (SR-500, Ver. 4.18B; SEED Technology, Bucheon, Republic of Korea). Trials with unnatural movement patterns or approach speed deviations were excluded and repeated until five valid trials were obtained (Figure 1).

### 2.3. Data Collection

Three-dimensional marker trajectories were collected using an eight-camera infrared motion capture system (Micus M3; Qualisys AB, Gothenburg, Sweden) at a sampling rate of 200 Hz, while GRF data were collected from a force plate (9260AA6, Kistler, Winterthur, Switzerland) at 1000 Hz. A modified Plug-in Gait model with 34 retroreflective markers (12.5 mm diameter) was applied to the trunk and lower-body segments—excluding head and upper extremities—to accommodate complex movements, with one additional marker on the IMU sensor for position tracking. The detailed configuration is shown in Figure 1.

IMU (Ultium EMG System, Noraxon, Scottsdale, AZ, USA) sampling at 200 Hz was positioned between the scapulae (approximately T4–T6 level) to replicate the typical Electronic Performance and Tracking Systems (EPTS) configuration [19]. The IMU comprised a triaxial accelerometer (±16 g), gyroscope (±2000°/s), and magnetometer (±4800 μT). The sensor coordinate system was remapped so that *X*-axis = ML (mediolateral), *Y*-axis = AP (anteroposterior), and *Z*-axis = vertical directions—applied consistently across all participants and used throughout this paper. All measurement systems were synchronized using Qualisys Track Manager software (version 2025.1; Qualisys AB, Gothenburg, Sweden).

### 2.4. Data Processing

#### 2.4.1. Data Preprocessing

Marker trajectory and force data were filtered using a fourth-order Butterworth low-pass filter with cut-off frequencies of 6 Hz and 30 Hz, respectively. Raw IMU signals were filtered at 20 Hz, followed by the application of a quaternion-based EKF. The analysis window was defined as the stance phase, from initial contact to toe-off, identified using a 20 N vertical GRF threshold. Inverse dynamics were applied to a lower-limb model in Visual3D (Version 2023.01.1, C-Motion, Kingston, ON, Canada) to calculate ankle and knee joint moments. Joint moments were defined according to their respective planes of motion: knee (sagittal: flexion/extension; frontal: valgus/varus; transverse: internal/external rotation) and ankle (sagittal: dorsiflexion/plantarflexion; frontal: inversion/eversion; transverse: internal/external rotation). To account for anthropometric differences, vGRF was normalized to each participant’s body weight (BW), and joint moments were normalized to body mass (Nm/kg). All data were time-normalized to 0–100% of the stance phase for model training and validation (Figure 1).

#### 2.4.2. Extended Kalman Filter (EKF)

**Figure 2:**
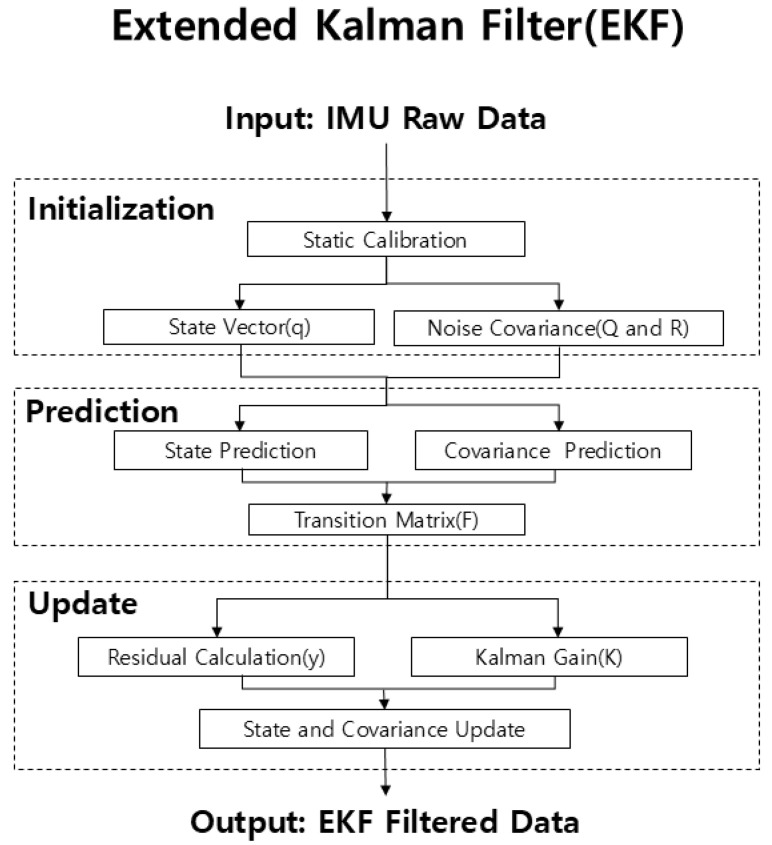
Flowchart of the Extended Kalman Filter (EKF) algorithm.

An EKF was implemented to correct nonlinear errors and achieve precise sensor orientation estimation from IMU data (Figure 2). As the development of a novel filtering algorithm was not the primary contribution of this work, we adopted an established and well-documented EKF framework widely utilized in IMU-based biomechanical research [14,20,21]. Previous studies have validated that this approach effectively minimizes sensor noise and integration drift, ensuring the reliability of the orientation data used as inputs for the subsequent kinetic prediction model. The algorithm operated at 200 Hz (△*t* = 0.005 s), with the system state vector X defined as a unit quaternion *q*. Gyroscope bias was estimated and removed during a static calibration period prior to data collection. The filter comprised two main steps

(1) $$X={\left[q_{0,}q_{1,}q_{2,}q_{3}\right]}^{T}$$

1. Prediction step: Angular velocity was used to estimate the next state orientation and error covariance *P*. 4 × 4 process noise covariance matrix *Q* accounted for model uncertainty, with *P* predicted as follows

(2) $${Ρ}_{k|k-1}={F_{k-1}P}_{k-1|k-1}F_{k-1}^{T}+Q$$

2. Update step: The accelerometer gravity vector (g = 9.80655 m/s^2^) served as a reference to compute the Kalman gain *K* and update the predicted state. Accelerometer uncertainty was modeled with a 3 × 3 measurement noise covariance matrix *R*. The corrected quaternions were converted to Euler angles (Roll, Pitch, Yaw) for use as prediction model input features.

(3) $$K_{k}=P_{k|k-1}H_{k}^{T}{\left(H_{k}P_{k|k-1}H_{k}^{T}+R\right)}^{-1}$$

(4) $${\hat{X}}_{k|k}=X_{k|k-1}+K_{k}y_{k}$$

### 2.5. Model Development

#### 2.5.1. Feature Extraction and Data Splitting

Twelve input features were used for model development, including nine primary IMU signals (three-axis acceleration, three-axis angular velocity, and three Euler angles) and three derived features (acceleration magnitude, angular velocity magnitude, and tilt angle). To quantify the dynamic inclination of the upper body, the tilt angle was defined as the root sum of squares of the roll and pitch angles

$$Tilt\;Angle=\sqrt{{roll}^{2}+{pitch}^{2}}$$

The dataset was split into the training set (80%) and test sets (20%) using a trial-based GroupShuffleSplit method. This approach was adopted to prevent underfitting given the limited sample size while ensuring the independence of the datasets by preventing time-series leakage [4,22]. By splitting the data at the trial level, the model could effectively learn intra-individual movement variability and player-specific strategies while maintaining strict separation between the training and test trials. All derived features were calculated individually after the split to ensure data integrity.

#### 2.5.2. Model Training and Hyperparameter Optimization

A Random Forest (RF) ensemble regression model from the Scikit-learn library was used to predict vGRF and lower-limb joint moments. Hyperparameters were optimized using RandomizedSearchCV with 5-fold cross-validation on the training set. The hyperparameter tuning was performed using a random search within the following predefined space: n_estimators {100, 150, 200, 250, 300}, max_depth {8, 10, 12, 15, 20}, min_samples_split {2, 5, 10}, min_samples_leaf {1, 2, 4}, and max_features {‘sqrt’, ‘log2’}. Based on this search space, the final optimized hyperparameters for each target variable are presented in Table 1.

### 2.6. Statistical Analysis and Model Validation

Model performance verification and statistical analysis were performed in a Python (version 3.12) environment using Scikit-learn for the coefficient of determination (*R*^2^) and root mean square error (RMSE), and SciPy library for Spearman’s rank correlation coefficient (*r*). All statistical analyses were conducted using non-parametric methods (*α* = 0.05). Feature importance was quantified using the Mean Decrease Impurity (MDI) method, based on the accumulated reduction in Mean Squared Error (MSE) contributed by each variable. Additionally, Bland–Altman analysis and 95% limits of agreement (LoA) were calculated to quantitatively assess bias and precision. Waveform similarity between predicted and reference time-series data was quantified using the coefficient of multiple correlation (CMC) calculated throughout the entire stance phase [23,24]. Interpretation of CMC values was based on established classification criteria: excellent (0.95–1.00), very good (0.85–0.95), good (0.75–0.85), moderate (0.65–0.75), and poor (<0.65) [25,26]. Quantitative analysis was supplemented with visual comparison of mean patterns. 

## 3. Results

### 3.1. Model Prediction Performance

The developed predictive model demonstrated satisfactory prediction accuracy for vGRF and sagittal plane joint moments. The vGRF showed high precision (*R*^2^ = 0.766; RMSE = 0.428 N/BW; *r* = 0.796), followed by strong performance in ankle and knee sagittal moments (*R*^2^ > 0.60; RMSE < 0.65 Nm/kg; *r* > 0.80). The ankle frontal moment also exhibited relatively good predictive power (*R*^2^ = 0.638; RMSE = 0.255 Nm/kg; *r* = 0.820), comparable to sagittal plane variables. In contrast, accuracy was lower for the remaining non-sagittal moments (*R*^2^ = 0.301–0.536; RMSE = 0.196–0.370 Nm/kg; *r* = 0.553–0.743) (Table 2). These results indicate a limited capacity to capture complex non-sagittal loading patterns. All correlations were statistically significant (*p* < 0.001).

### 3.2. Waveform Analysis

**Figure 3:**
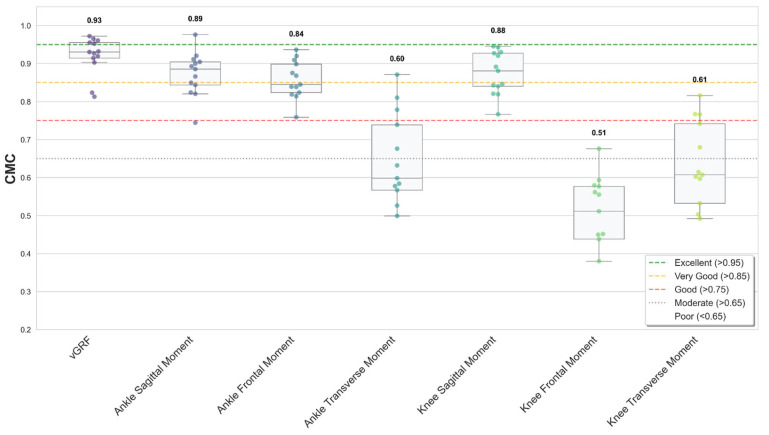
Distribution of CMC values for vGRF and lower-limb joint moments [23]. Boxes represent the interquartile range (IQR), horizontal lines indicate median values, whiskers extend to 1.5 × IQR, and individual trials are shown as dots. Dashed dotted lines represent thresholds for excellent (>0.95, green), very good (>0.85, orange), and good (>0.75, red), moderate (>0.65, gray dotted), and poor (<0.65) waveform [25,27].

**Figure 4:**
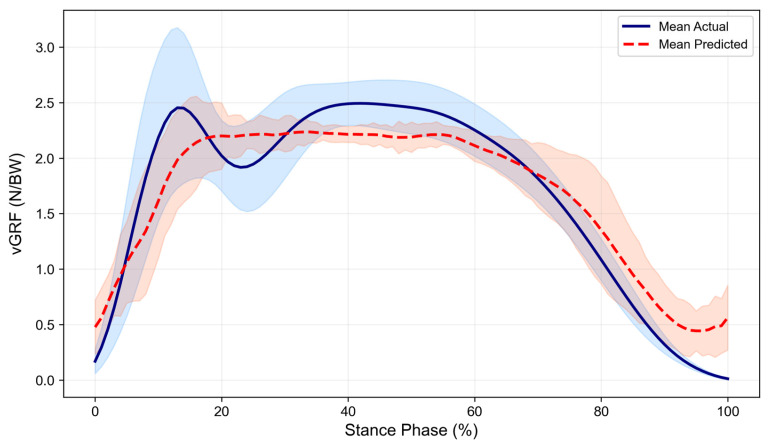
Time-series comparison between actual and predicted vGRF values during the stance phase of cutting. Solid and dashed lines represent the mean values, and shaded areas indicate the standard deviation (±SD). vGRF data are normalized to body weight (BW).

**Figure 5:**
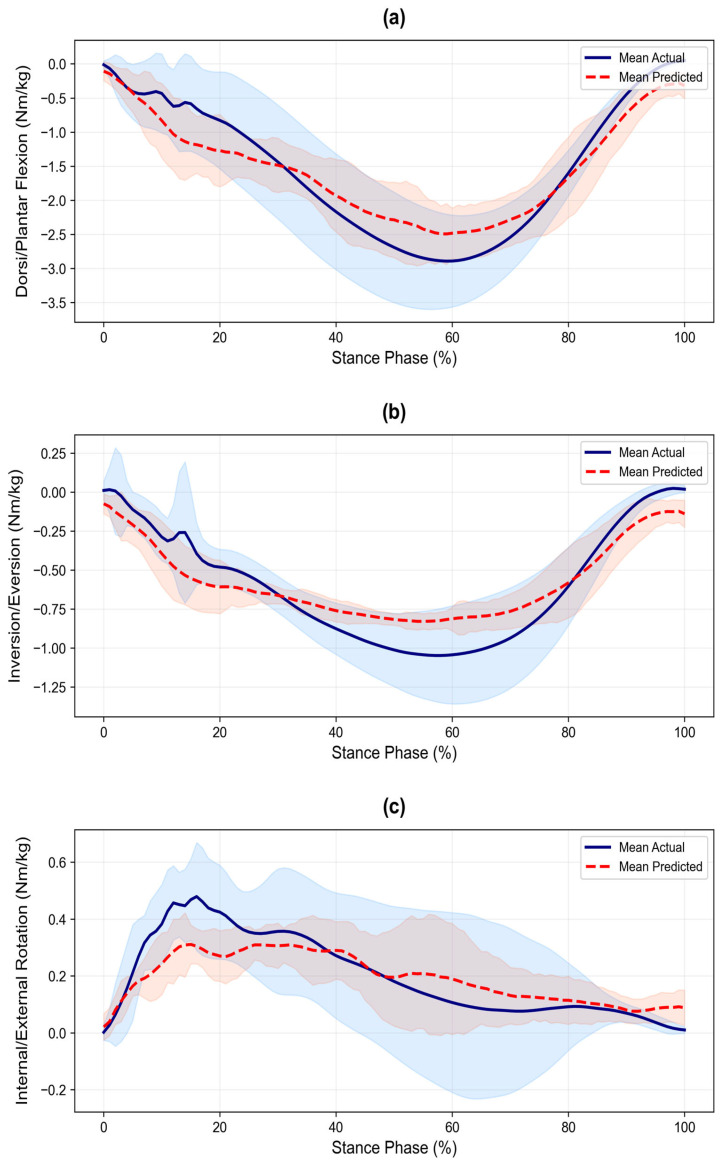
Time-series comparison of ankle moments between actual and predicted values during the stance phase of cutting: (**a**) Sagittal plane moment (Dorsi/Plantarflexion), (**b**) Frontal plane moment (Inversion/Eversion), and (**c**) Transverse plane moment (Internal/External Rotation). Solid and dashed lines represent the mean values, and shaded areas indicate the standard deviation (±SD). All joint moments are normalized to body mass (kg).

**Figure 6:**
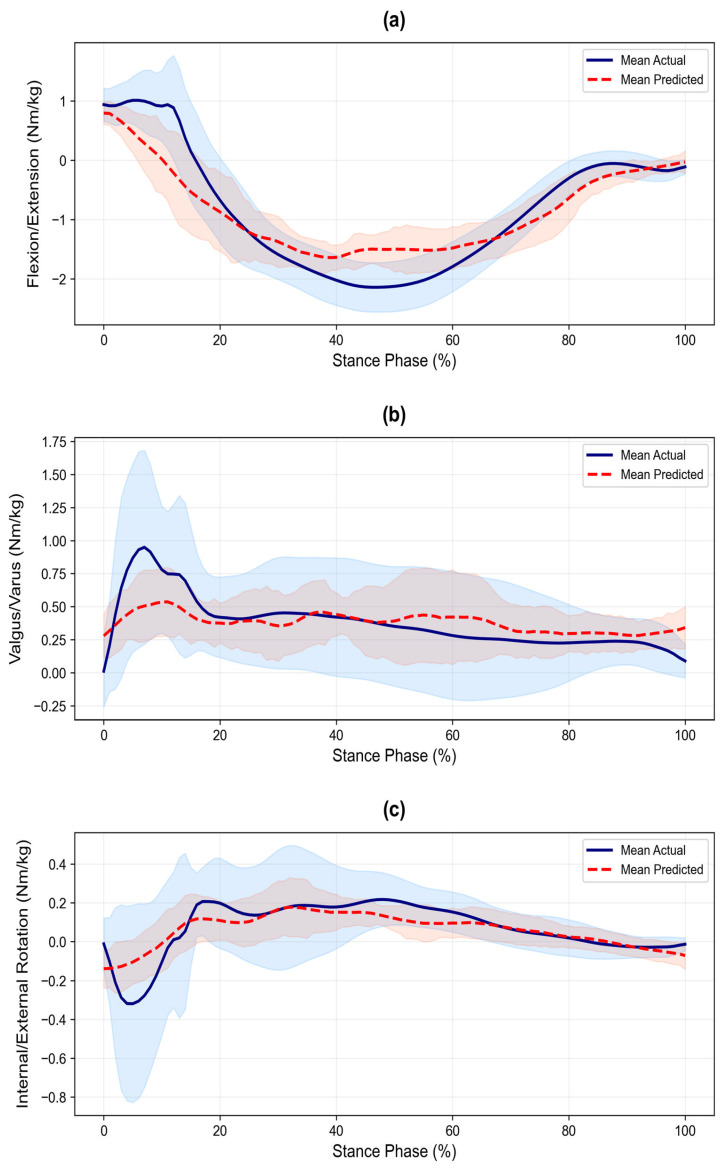
Time-series comparison of knee moments between actual and predicted values during the stance phase of cutting: (**a**) Sagittal plane moment (Flexion/Extension), (**b**) Frontal plane moment (Valgus/Varus), and (**c**) Transverse plane moment (Internal/External Rotation). Solid and dashed lines represent the mean values, and shaded areas indicate the standard deviation (±SD). All joint moments are normalized to body mass (kg).

Waveform similarity was quantified using the CMC (Figure 3). Very good agreement (median > 0.85) was achieved for vGRF (median = 0.93), ankle sagittal (0.89), and knee sagittal (0.88) moments. The ankle frontal moment showed good agreement (0.84). In contrast, ankle transverse (0.60), knee frontal (0.51), and knee transverse (0.61) moments showed poor agreement (median < 0.65), indicating limited waveform similarity in non-sagittal planes. Mean waveforms (Figure 4, Figure 5 and Figure 6, smoothed with a 5-point moving average for visual presentation) were presented for qualitative assessment. Across all planes, the predicted trajectories generally followed the temporal and functional trends of the measured data.

However, the model showed a consistent tendency toward smoothing, resulting in more conservative trajectories with reduced amplitude variability. Specifically, the predicted waveforms exhibited a more gradual slope during the initial loading phase and a flattened appearance compared to the reference data, particularly in non-sagittal planes. While the general movement trends were maintained, these smoother curves did not fully replicate the fluctuations observed in the actual measurements.

### 3.3. Agreement Analysis

As shown in Table 3, bias for all variables in the cutting condition ranged from −0.066 to 0.044 Nm/kg, indicating minimal average difference between predicted and actual values. However, the LoA width exhibited a distinct gradient across variables. The widest bands were observed for sagittal moments (LoA width ≈ 2.5), followed by vGRF (LoA width ≈ 1.6). In contrast, the non-sagittal moments showed the narrowest bands, with widths below 1.0.

### 3.4. Feature Importance Analysis

Acceleration-based features contributed most significantly to the prediction of vGRF and sagittal plane moments. Specifically, Acceleration Magnitude (27.30%) and AP Acceleration (26.44%) were the dominant predictors for vGRF. In contrast, for frontal and transverse plane moments, the model utilized distinct kinematic features; notably, ML Angular Velocity (17.94%) was the top predictor for the ankle transverse moment, while ML Acceleration (10.97%) showed a meaningful contribution to the ankle frontal moment. This indicates that the model distinguishes and utilizes specific sensor signal types based on the movement plane of the target variable (Table 4).

**Table 3:** Bland–Altman agreement analysis between predicted and actual values by variable.

Variable	Bias	95% LoA
vGRF(N/BW)	−0.004	[−0.811, 0.794]
Ankle Sagittal Moment (Nm/kg)	−0.066	[−1.189, 1.276]
Ankle Frontal Moment (Nm/kg)	−0.004	[−0.416, 0.534]
Ankle Transverse Moment (Nm/kg)	−0.004	[−0.412, 0.400]
Knee Sagittal Moment (Nm/kg)	0.006	[−1.340, 1.188]
Knee Frontal Moment (Nm/kg)	0.044	[−0.845, 0.636]
Knee Transverse Moment (Nm/kg)	−0.034	[−0.398, 0.482]

Note. Bias represents the mean difference between predicted and actual values; values closer to zero indicate less systematic error. LoA (Limits of Agreement) denotes ±1.96 standard deviations of the mean difference; narrower LoA widths indicate higher prediction precision. vGRF = vertical ground reaction force.

**Table 4:** Feature importance analysis results: top 3 contributing features by variable.

Target Variable	Feature	Contribution (%)
vGRF	Acceleration magnitude	27.30
	AP Acceleration	26.44
	Vertical Acceleration	9.87
Ankle Sagittal Moment	Acceleration magnitude	18.86
	Vertical Acceleration	15.05
	AP Acceleration	11.66
Ankle Frontal Moment	Acceleration magnitude	27.91
	AP Acceleration	21.09
	ML Acceleration	10.97
Ankle Transverse Moment	ML Angular Velocity	17.94
	Acceleration magnitude	10.50
	Vertical Acceleration	9.51
Knee Sagittal Moment	Acceleration magnitude	22.23
	AP Acceleration	17.91
	ML Acceleration	17.28
Knee Frontal Moment	AP Acceleration	16.29
	ML Angular Velocity	15.99
	Vertical Angular Velocity	14.81
Knee Transverse Moment	AP Acceleration	18.56
	Acceleration magnitude	12.67
	Vertical Angular Velocity	12.07

Note. AP = anteroposterior; ML = mediolateral. Acceleration magnitude and angular velocity magnitude represent the resultant vectors calculated from triaxial sensor data. vGRF = vertical ground reaction force.

## 4. Discussion

In this study, the proposed approach effectively predicted vGRF and sagittal moments from trunk-mounted IMU data during football cutting, while revealing single-sensor limitations for non-sagittal moments

### 4.1. Biomechanical Validity of Trunk-Mounted IMU

The capacity to estimate lower-limb kinetics from trunk-mounted sensor data is grounded in top-down inverse dynamics principles. Trunk-mounted acceleration and inertial dynamics, representing approximately 60% of total body mass, exert a dominant influence on GRF generation and lower-limb joint loading. The feature importance analysis identified vertical and AP accelerations as primary predictors, demonstrating that the model effectively captures the fundamental mechanical relationship (*F* = *ma*) between center-of-mass acceleration and external forces. These findings biomechanically validate trunk-mounted IMUs as reliable surrogate variables for whole-body kinetics. This is consistent with recent findings demonstrating that upper-body acceleration is an essential component for estimating lower-limb joint moments [28].

### 4.2. Kinematic Constraints of Non-Sagittal Prediction

A prominent limitation of this study was the attenuated prediction performance for non-sagittal variables. The narrower LoA widths (<1.0) despite low *R*^2^ suggest conservative precision around mean patterns, corroborated by poor CMC fidelity and waveform smoothing (Figure 4, Figure 5 and Figure 6). The difficulty in predicting these 3D rotational loads using trunk-mounted IMU data alone arises from the kinematic dissociation between upper and lower body segments during cutting. Pelvic and thoracic rotations interact complexly with lower-limb joint loading during high-risk cutting motions [29]. Although our model showed high reliance on angular velocity signals for non-sagittal predictions, the single trunk-mounted sensor provided insufficient information resolution to fully capture subtle lower limb torsional dynamics. Notably, while the model successfully utilized ML Angular Velocity (17.94%) for the Ankle Transverse moment, it still relied heavily on AP Acceleration for the Knee Transverse moment (18.56%). This indicates that even with angular velocity features, a single trunk sensor struggles to distinguish between whole-body deceleration and specific knee rotational loading. This underscores the physical constraints of single-sensor-based predictions in capturing closed-kinetic-chain rotational dynamics.

The fundamental limitations of this study are the reduced predictive performance for non-sagittal plane moments and the systematic underestimation of rapid peak loads during landing impacts. The poor performance in non-sagittal planes primarily stems from the physical constraints of a single trunk-mounted IMU, as multi-planar kinematics are highly susceptible to signal attenuation along the biomechanical chain. Furthermore, regression tree ensemble algorithms inherently smooth extreme values; consistent with similar machine learning-based biomechanical studies, peak values were underestimated by approximately 17% [4]. Since peak multi-planar loads—such as knee valgus and internal rotation moments—are primary determinants of the tissue failure thresholds underlying ACL injury, the current model is not suited for direct diagnosis of specific acute injury events. A quantitative analysis establishing specific injury thresholds based on estimated loads remains an essential objective for future research.

Despite these limitations, the model offers meaningful practical utility when integrated into a tiered monitoring system. Practitioners can use the reliable vGRF and sagittal plane data as primary screening indicators to track cumulative mechanical loads across training sessions, monitor relative load distribution among teammates, and detect fatigue-related changes in loading patterns. For players exhibiting persistently abnormal cumulative load profiles, a stepped approach—classifying the athlete as at risk and recommending a secondary precision biomechanical evaluation—is both feasible and clinically meaningful. Future research should pursue data augmentation via OpenSim musculoskeletal simulations and integration of temporal architectures such as LSTM or Transformer models to improve predictive accuracy for non-sagittal moments and peak impact loads.

### 4.3. Extended Kalman Filter and Sensor Fusion Scalability

This study implemented an EKF to remove IMU sensor noise and enable quaternion-based 3D orientation estimation. A key practical strength lies in data collection at FIFA EPTS standard mounting locations. Since all EPTS systems generate IMU data from identical positions, the proposed prediction algorithm is directly applicable to existing systems. Additionally, GNSS fusion compensates for sensor drift to ensure long-term monitoring accuracy [14,30]. This demonstrates the potential of the proposed kinetic prediction algorithm to scale from laboratory prototypes to commercial EPTS solutions capable of simultaneous field-based kinetic and kinematic monitoring in match environments.

### 4.4. Machine Learning Model Selection and Performance Characteristics

Although the Random Forest algorithm is inherently non-temporal, it achieved reasonable predictive accuracy for vGRF and sagittal plane joint moment trajectories in this study [31]. We attribute this, in part, to the time-normalization of stance phase data, which aligns key biomechanical events across trials and implicitly supplies the model with phase-related temporal context—effectively reducing the difficulty of the prediction task, consistent with previous IMU-based kinetic prediction studies [32,33].

That said, this approach carries inherent limitations. Because time-normalization itself introduces implicit temporal information, the performance figures reported here may be somewhat optimistic relative to a scenario in which raw time-series data are used without normalization. Beyond this, the model’s reliance on a non-temporal architecture meant that high-frequency signal dynamics were not fully captured, leading to systematic underestimation of sharp transient loads during the initial contact phase—a pattern consistent with known limitations of ensemble regression methods [33]. Future work should therefore explore temporal architectures such as LSTM networks or Transformers, particularly for applications where accurate peak load estimation is critical [29,32].

The proposed framework is intended for post hoc kinetic load monitoring using IMU data collected in the field. For practical deployment, the stance phase can be automatically segmented using existing IMU-based gait event detection algorithms, which have been validated to identify initial contact and toe-off within ±10–20 ms at 200 Hz [34,35,36]. That said, even within this tolerance, a ±10 ms detection error translates to approximately ±4–5 percentage points of phase displacement on the time-normalized axis—given the mean stance duration of 200–250 ms in our cutting trials. This degree of temporal misalignment is particularly consequential during the early stance phase (0–20%), where transient peak loads are concentrated, and is expected to have a larger relative impact on non-sagittal plane moments, which already show limited predictive accuracy. Future work should develop an integrated pipeline combining automatic gait event detection with the kinetic prediction model, and empirically quantify the sensitivity of prediction accuracy to stance detection error.

### 4.5. Limitations

Although this study adopted a standardized 45-degree cutting task to establish the foundation for a single-IMU-based prediction model, the players’ inherent cutting strategies and landing patterns were not artificially constrained, thereby preserving a degree of ecological validity within the experimental design.

The present study has several limitations that warrant consideration. The sample was restricted to 13 healthy male amateur players, which limits the diversity of training data and may reduce model performance in populations with different biomechanical profiles. Female athletes tend to exhibit greater knee valgus angles and reduced knee flexion during cutting maneuvers [2,37], characteristics associated with higher ACL injury rates, and were not represented in the current dataset. Expanding the cohort to include female athletes and players of varying competitive levels should be a priority in future work.

The trial-based GroupShuffleSplit validation scheme adopted here may produce overly optimistic performance estimates, given that trials from the same participant appear in both training and test sets [4,22,38]. Under a subject-independent scheme such as leave-one-subject-out cross-validation, predictive accuracy would likely be lower. Future studies should validate the model against truly unseen participants before field deployment is considered.

Finally, the protocol was limited to a single 45° cutting angle at a controlled approach speed. Match-play cutting involves a considerably wider range of directions, speeds, and anticipatory demands, and it remains unclear whether the model’s performance would hold under such conditions. Validation across varied cutting tasks is needed before broader application can be supported.

## 5. Conclusions

This study presents a practical EPTS-compatible technology that extends laboratory-grade biomechanical analysis to match environments by effectively predicting vGRF and sagittal joint moments during football cutting using a single IMU at EPTS standard locations and random forest modeling. This enables coaches and sports scientists to perform field-based kinetic load monitoring, supporting data-driven player management and injury risk mitigation.

## Figures and Tables

**Table 1:** Optimal hyperparameters for the Random Forest model.

Target Variable	n_estimators	max_depth	min_samples_split	min_samples_leaf	max_features
vGRF	250	10	2	2	log2
Ankle Sagittal Moment	100	15	10	1	log2
Ankle Frontal Moment	100	20	10	4	log2
Ankle Transverse Moment	200	20	10	4	log2
Knee Sagittal Moment	200	10	5	2	log2
Knee Frontal Moment	300	15	2	2	log2
Knee Transverse Moment	100	20	10	2	log2

Note: vGRF = vertical Ground Reaction Force. The hyperparameters (n_estimators, max_depth, min_samples_split, min_samples_leaf, max_features) follow the standard terminology of the Random Forest algorithm.

**Table 2:** Prediction performance for cutting.

Variable	*R* ^2^	RMSE	*r*
vGRF(N/BW)	0.766	0.428	0.796 ***
Ankle Sagittal Moment (Nm/kg)	0.689	0.647	0.842 ***
Ankle Frontal Moment (Nm/kg)	0.638	0.255	0.842 ***
Ankle Transverse Moment (Nm/kg)	0.374	0.196	0.591 ***
Knee Sagittal Moment (Nm/kg)	0.661	0.649	0.807 ***
Knee Frontal Moment (Nm/kg)	0.348	0.370	0.562 ***
Knee Transverse Moment (Nm/kg)	0.301	0.214	0.593 ***

Note. *R*^2^ = coefficient of determination; RMSE = root mean square error; *r* = Spearman’s rank correlation coefficient between predicted and actual values. *** *p* < 0.001. vGRF = vertical ground reaction force. *R*^2^ performance criteria per previous studies: >0.8 = excellent; >0.6 = good; >0.4 = moderate; ≤0.4 = limited [27].

## Data Availability

Dataset available on request from the authors.
